# A pivotal role for the IL-1β and the inflammasome in preterm labor

**DOI:** 10.1038/s41598-024-54507-w

**Published:** 2024-02-20

**Authors:** T. E. Lopez, H. Zhang, E. Bouysse, F. Neiers, X. Y. Ye, C. Garrido, M. Wendremaire, Frédéric Lirussi

**Affiliations:** 1 INSERM U1231, Labex LIPSTIC and Label of Excellence from la Ligue Nationale Contre le Cancer, 21000 Dijon, France; 2 Faculty of Medicine and Pharmacy, University of Burgundy, 21000 Dijon, France; 3 School of Pharmacy, Hangzhou Normal University, Hangzhou, Zhejiang China; 4 Cancer Center George-François Leclerc, 21000 Dijon, France; 5 Laboratory of Pharmacology-Toxicology, Platform PACE, University Hospital Besançon, 25000 Besançon, France; 6 Faculty of Medicine and Pharmacy, University of Franche-Comté, 25000 Besançon, France

**Keywords:** Reproductive biology, Mechanisms of disease, Inflammasome

## Abstract

During labor, monocytes infiltrate massively the myometrium and differentiate into macrophages secreting high levels of reactive oxygen species and of pro-inflammatory cytokines (i.e. IL-1β), leading to myometrial contraction. Although IL-1β is clearly implicated in labor, its function and that of the inflammasome complex that cleaves the cytokine in its active form, has never been studied on steps preceding contraction. In this work, we used our model of lipopolysaccharide-induced preterm labor to highlight their role. We demonstrated that IL-1β was secreted by the human myometrium during labor or in presence of infection and was essential for myometrial efficient contractions as its blockage with an IL-1 receptor antagonist (Anakinra) or a neutralizing antibody completely inhibited the induced contractions. We evaluated the implication of the inflammasome on myometrial contractions and differentiation stages of labor onset. We showed that the effects of macrophage-released IL-1β in myometrial cell transactivation were blocked by inhibition of the inflammasome, suggesting that the inflammasome by producing IL-1β was essential in macrophage/myocyte crosstalk during labor. These findings provide novel innovative approaches in the management of preterm labor, specifically the use of an inflammasome inhibitor to block the precursor stages of labor before the acquisition of the contractile phenotype.

## Introduction

Preterm labor (PTL) occurs before 37 weeks of gestation and its prevalence rises in industrialized countries despite advancing knowledge of related mechanisms and risks factors^1^. With approximately 15 million of premature births, PTL remains a major obstetric challenge and represents 75% of perinatal mortality and more than half of long-term morbidity^2^. If many cases of spontaneous PTL are unexplained, a significant proportion (40 up to 70%) is linked to genital tract infection or chorioamnionitis^2,3^. Genesis of term and preterm labor is strongly correlated with the inflammatory cascade of events physiologically activated at term but also pathologically activated preterm^4–6^. Labor involves a transition to a contractile phenotype characterized by differentiation stages including cytoskeleton reorganization and synchronization of myometrial cells^7^. Understanding the mechanisms of term labor may then lead to identify therapeutic targets for the management of preterm labor.

It is established that the inflammatory event, implicated in labor onset, results from the massive infiltration of primed macrophages and neutrophils into the myometrium^8,9^ secreting inflammatory messengers (i.e., reactive oxygen species (ROS), cytokines, prostaglandins) and inducing the expression of specific labor markers^10,11^. Previous data from our team highlighted the macrophage’s role in myometrial cells activation^12,13^. We demonstrated that, in laboring myometrium, ROS level was increased as a result of their production by macrophages^12^. Our co-culture model of primary human macrophages and myometrial cells confirmed ROS as major intermediate messengers during labor^13^. Pro-inflammatory cytokines have also been identified in labor induction^14^. These mediators are then produced by leukocytes during term labor and at an even higher level during preterm, particularly in the presence of infection (i.e., chorioamnionitis)^11,15–17^. Among others, IL-1β has been shown to be implicated in labor onset by inducing uterine activation proteins expression^18^, progesterone metabolism^19^ and oxytocin secretion and an up-regulation of its receptor^20,21^. Furthermore, in case of sterile inflammation, high concentration of IL-1 β in the serum or the amniotic fluid, is correlated with PTL^22,23^ and in vivo administration of IL-1β on animal models induces parturition within 48 h^23–25^.

IL-1β is expressed in an inactive conformation, pro-IL-1β which is processed to its active and secreted form by the activated caspase-1 protein which is a part of the multiprotein inflammasome complexes^26,27^. Several canonical inflammasomes have been characterized, named according to the scaffold protein recruited^28^, and were activated by different mechanisms^29^. Inflammasomes are activated in response to microbial patterns or danger signals but their implication in the pathophysiological pathways of autoimmune or autoinflammatory diseases has also been described^30,31^. Recently, the level of macrophages expressing NLRP3 inflammasome was shown to be higher in peripheral blood of women with preterm premature rupture of membranes compared to normal full‐term pregnancies^32^. Furthermore, chemically or genetically deficiency of NLRP3 induces resistance to prematurity suggesting that inflammasome signaling plays a key role in PTL^33–35^. Furthermore, caspase-1 activation has also been associated with spontaneous human term labor in the myometrium and was required for LPS-induced IL-1β secretion^36^ but neither the cell types involved in IL-1β secretion, nor the molecular effects of IL-1β on myometrial cells were deciphered.

Since modulation of inflammasome activation and activity seems essential in the management of inflammatory pathologies such as preterm labor, in this work, we investigated the role of IL-1β in the contraction and differentiation steps (i.e. cytoskeleton reorganization and cell synchronization) of myometrial cells using an in vitro co-culture model of LPS-induced labor onset. This study also aimed to explore the potential therapeutic effects of the inhibition of IL-1β (i.e. Anakinra or neutralizing antibody) or inflammasome (i.e. MCC950).

## Results

### IL-1β is over-expressed during labor and is required for myometrial contractions

**Figure 1:**
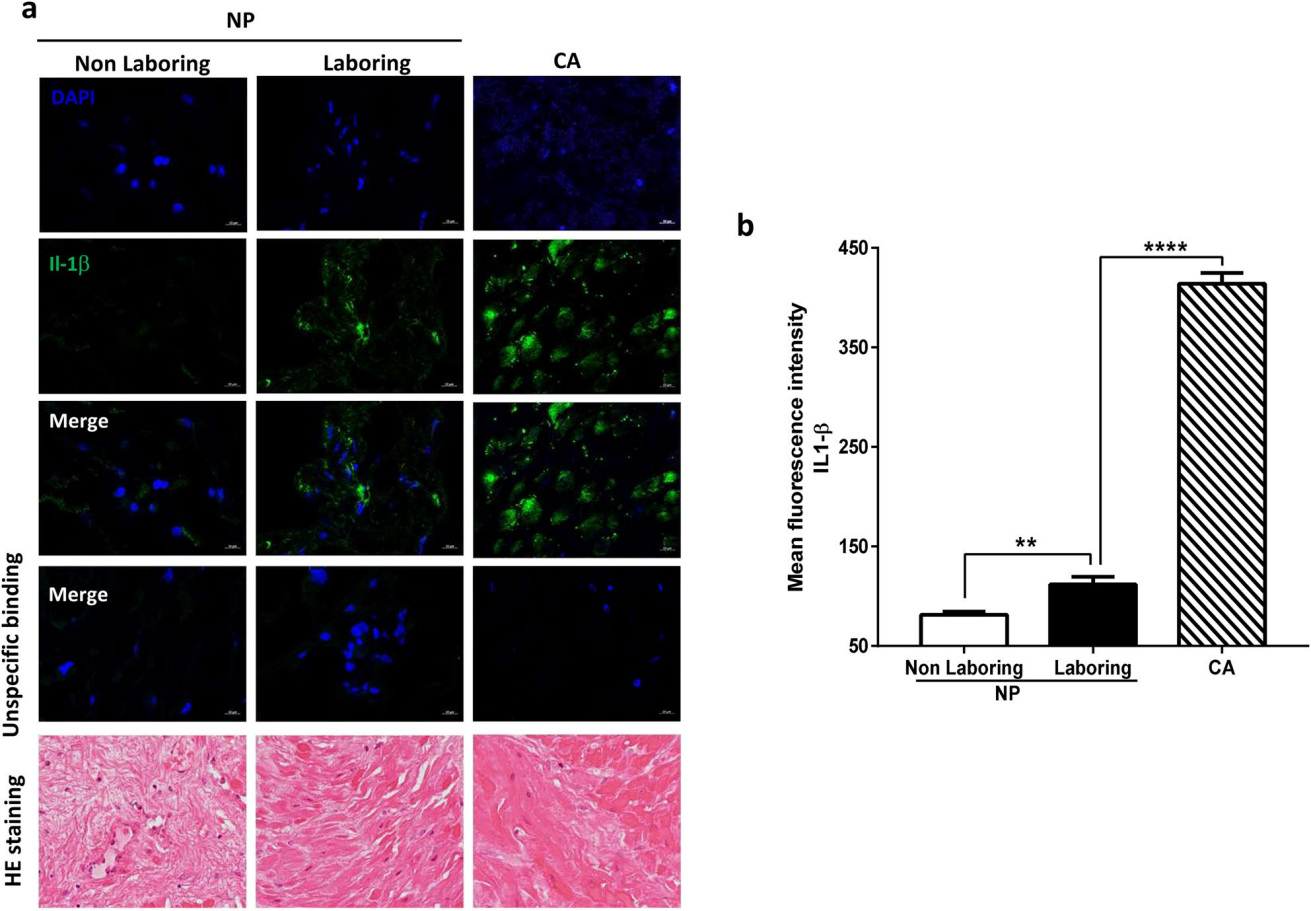
IL-1β is over-expressed in myometrium during labor. (**a**) Pictures of myometrium from non-laboring and laboring patients during normal pregnancy (NP) or with chorioamnionitis (CA). Images of IL-1β or hemalum eosine staining are representative of 5 pictures taken from 3 different samples for each condition with an Axiozoom epifluorescence microscope (×400) in random chosen fields. (**b**) Fluorescence intensity of IL-1β labeling, mean ± SEM, n = 15, **P < 0.01 and ****P < 0.0001.

We first assessed IL-1β expression in myometrial biopsies from normal pregnancy (NP) at term nonlaboring, term laboring and from patients with chorioamnionitis (CA). We observed that IL-1β expression was highly increased in the myometrium during spontaneous labor and even more in case of CA (Fig. 1a,b).

**Figure 2:**
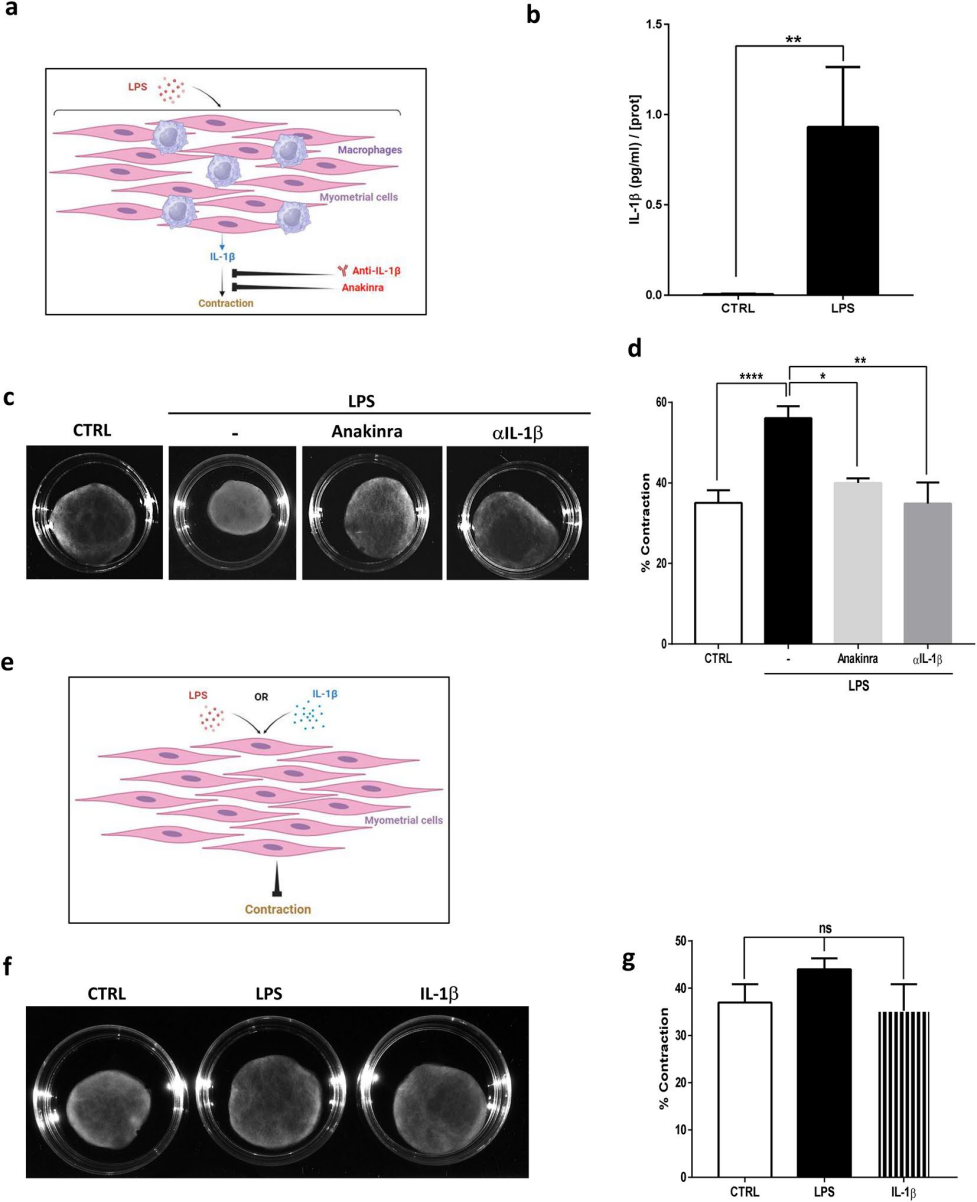
IL-1β is required for contractions of collagen lattices of primary human myometrial cells co-cultured with macrophages. (**a**) Schematic representation of the LPS-mediated IL-1β effects on myometrial cells contraction cultured with macrophages. (**b**) IL-1β secretion in co-culture cell medium. Macrophages and myometrial cells were cultured for 24 h in presence or absence of LPS (100 ng/ml). Supernatants were collected and IL-1β was evaluated with ELISA assay. Concentrations were standardized with protein cell content and presented as means ± SEM, n = 6, **P < 0.01. (**c**) Representative photographs of collagen lattices of myometrial cells co-cultured with macrophages stimulated with LPS (100 ng/ml), in presence or absence of Anakinra (10 µg/ml) or αIL-1β (1 µg/ml) at 96 h. (**d**) Mean percentage contraction of collagen lattices after 96 h of stimulation with LPS or LPS + Anakinra (10 µg/ml) or LPS + αIL-1β (1 µg/ml) compared to the basal surface area, mean ± SEM, n = 5, *P < 0.05, **P < 0.01 and ****P < 0.0001. (**e**) Schematic representation of the IL-1β effects on myometrial cells contraction. **f)** Photographs of collagen lattices of myometrial cells alone in presence of LPS (100 ng/ml) or IL-1β (1 ng/ml). (**g**) Mean percentage contraction of collagen lattices ± SEM after 96 h of LPS or IL-1β treatment, compared to the basal surface area.

We further characterized the implication of IL-1β during labor by using our in vitro labor model of human macrophages co-cultured with myometrial cells (Fig. 2a). We first measured the production of IL-1β in the co-culture supernatant and demonstrated that LPS stimulation induced IL-1β production (Fig. 2b).

Using collagen lattices, we evaluated the contribution of IL-1β in the contractile effect induced by LPS (055:B5) stimulation on myocytes and macrophages cultures. To do so, we used an IL-1β receptor antagonist (Anakinra) and an IL-1β neutralizing antibody (αIL-1β). LPS stimulation of collagen lattices of myocytes and macrophages induced a spontaneous myometrial contraction as we already reported^13^, and both the inhibition of IL-1β receptor by Anakinra or the neutralization of IL-1β by a specific antibody abrogated LPS-induced contraction (Fig. 2c,d). Indeed, in presence of these agents, the percentage of collagen contraction observed with LPS returned to the values obtained in the control condition (CTRL) (Fig. 2d).

Finally, we checked if IL-1β by itself was able to induce effective contractions on myometrial cells cultured alone. For this experiment, we seeded only myometrial cells in collagen lattices and stimulated them with LPS or IL-1β (Fig. 2e). We observed that, after 96 h of treatment, IL-1β, like LPS alone, failed to induce myocytes contraction (Fig. 2f,g). Taken together, these results indicate that IL-1β is required but is not sufficient for myocytes contraction.

### IL-1β has different effects on myometrial differentiation

**Figure 3:**
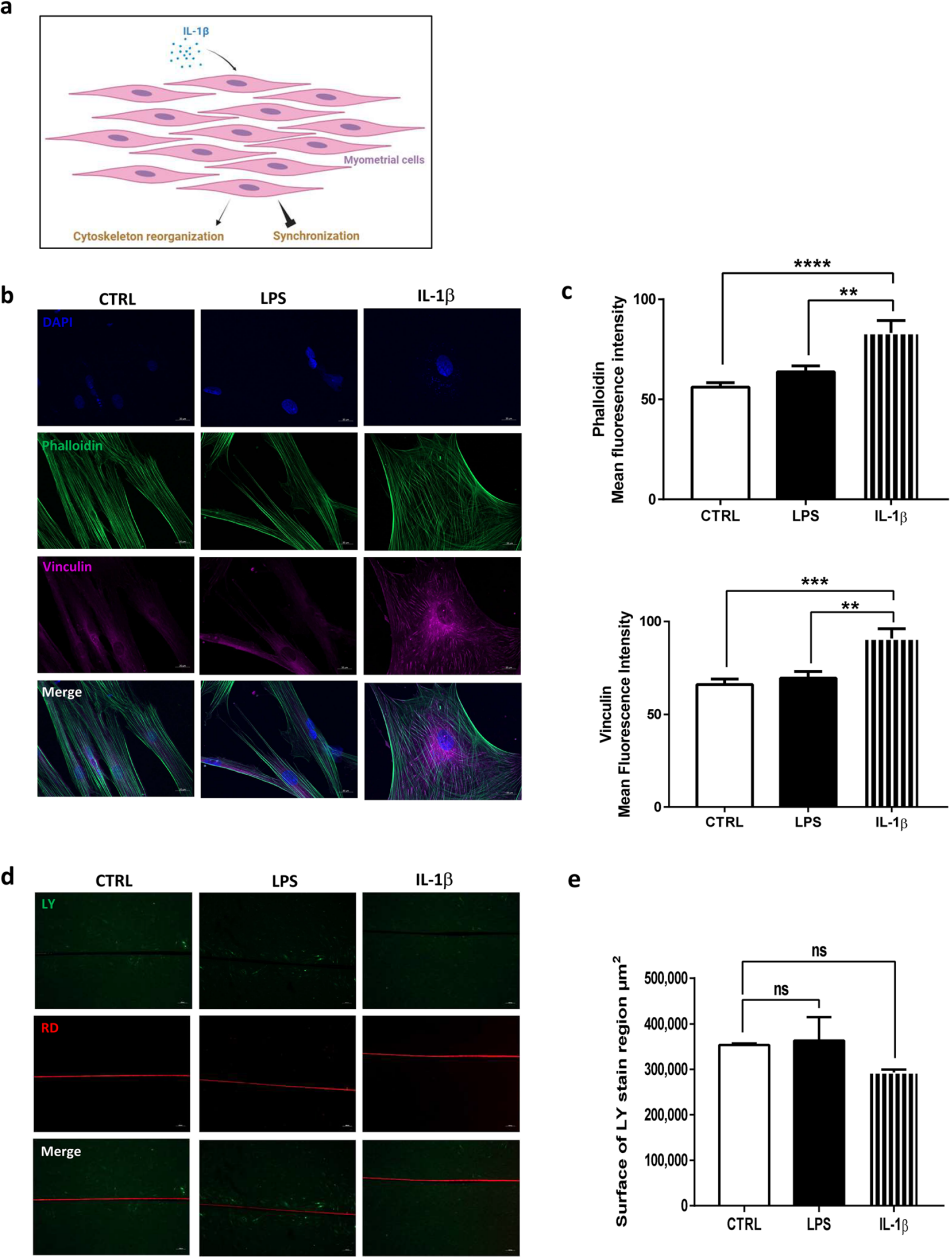
IL-1β has different effects on the differentiation of primary myometrial smooth muscle cells. (**a**) Schematic representation of IL-1β effects on differentiation of myometrial cells. (**b**) Myometrial cells were treated with LPS (100 ng/ml) or IL-1β (1 ng/ml) and stained with phalloidin (green), vinculin (purple) and DAPI (blue) for nuclear localization. Images, taken with an Axiozoom epifluorescence microscope (×400), are representative of 5 random pictures of four independent experiments. (**c**) Mean fluorescence intensity of phalloidin or vinculin ± SEM, n = 20, **P < 0.01, ***P < 0.001 and ****P < 0.0001. (**d**) Fluorescence images of LY (green) and RD (red) transfer to adjacent cells after scrap loading. Cells were treated for 24 h with LPS or IL-1β. Pictures were taken with an inverted microscope at ×100 magnification in two random chosen fields and are representative of three experiments. (**e**) Mean surface of LY staining ± SEM, n = 6.

Since labor and subsequently myometrial contractions are preceded by myometrial cell differentiation, we then evaluated the effect of IL-1β on cytoskeleton reorganization and functional gap junctions (GJ) formation on myometrial cells cultured alone (Fig. 3a). Cytoskeleton reorganization was assessed by staining of actin fibers and the anchoring vinculin protein. Unlike LPS, IL-1β stimulation by itself modified cell morphology in triangular-shape, representative of the in vitro laboring myocyte profile described previously^13,37,38^ (Fig. 3b). Actin fibers were extended to the entire cell length and colocalized with vinculin, indicating focal adhesion sites formation. Furthermore, mean fluorescence intensity measurement revealed that IL-1β stimulation induced actin fibers and vinculin synthesis, essential for myometrial cell remodeling (Fig. 3c). Finally, we investigated functional GJ formation between myocytes, a major step for effective myometrial contraction. By using the GJ permeable fluorescent dye Lucifer Yellow (LY), we evaluated GJ intercellular communication by the Scrape Loading/Dye Transfer assay (SL/DT assay). The transfer of lucifer yellow in adjacent cells allows to conclude to intercellular communication while no fluorescence indicates the absence of communication. As shown in Fig. 3d, IL-1β failed to induce LY transfer to adjacent cells via open GJ channel. The cell layer confluence was evaluated with phase contrast images (Supplemental Fig. 1a). Quantification of LY diffusion within the cell layer was not different whether or not myometrial cells were treated with LPS or IL-1β (Fig. 3e).

These results demonstrated that myometrial stimulation by IL-1β leads to structural modifications without using cellular synchronization, making it difficult to generate effective contractions.

### Blockade of LPS-induced IL-1β prevents myometrial differentiation

**Figure 4:**
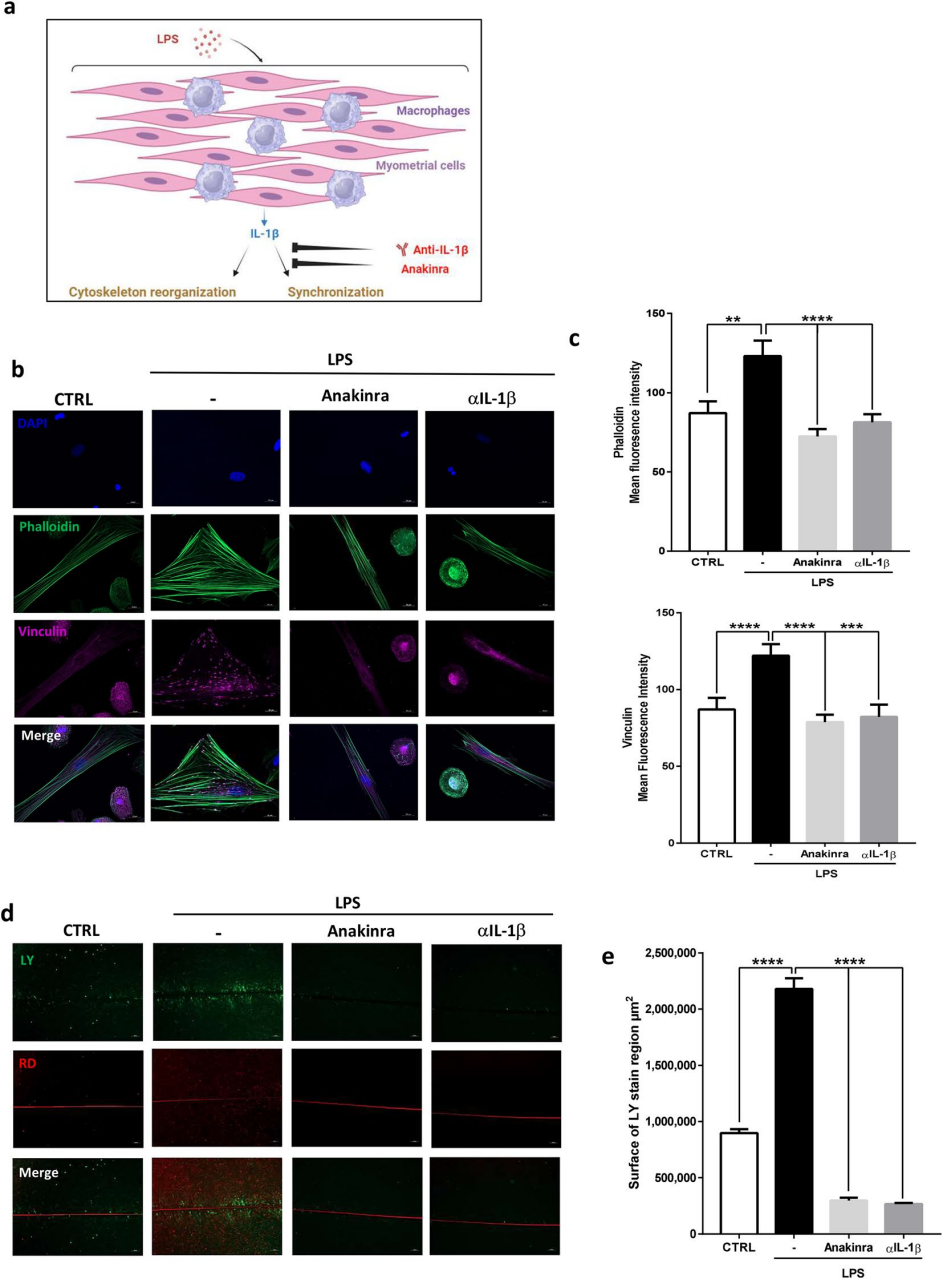
Neutralizing IL-1β effect prevents the differentiation of primary human myometrial smooth muscle cells. (**a**) Schematic representation of the LPS effects on differentiation of myometrial cells in a co-culture model. (**b**) Representative images of myometrial cells and macrophages co-cultures treated with LPS (100 ng/ml) in presence or absence of Anakinra (1 µg/ml) or αIL-1β (100 ng/ml) and stained with phalloidin (green), vinculin (purple) and DAPI (blue) for nuclear localization. Images, taken with an Axiozoom epifluorescence microscope (×400), are representative of 5 random pictures of four independent experiments. (**c**) Mean fluorescence intensity of phalloidin or vinculin ± SEM, n = 20, **P < 0.01, ***P < 0.001, ****P < 0.0001. (**d**) Fluorescence images of LY (green) and RD (red) transfer to adjacent cells after scrap loading. Cells were treated for 24 h with LPS ± Anakinra or αIL-1β. Pictures were taken with an inverted microscope at ×100 magnification in two random chosen fields and are representative of three experiments. (**e**) Mean surface of LY staining ± SEM, n = 6, ****P < 0.0001.

Since IL-1β by itself only lead to partial differentiation of myometrial cells alone, we investigated whether this cytokine was nevertheless necessary for this process in a co-culture model. For that, we studied the effect of IL-1β blockage on myometrial cells differentiation when cultured with macrophages (Fig. 4a). First, we evaluated cytoskeleton reorganization using the in vitro co-culture model of myometrial cells and macrophages (Supplemental Fig. 2). We showed that LPS stimulation led to myometrial cell remodeling which was fully abolished by the addition of Anakinra or αIL-1β. Indeed, under these conditions, we noticed that actin fibers remained parallel along the cells and were no longer colocalized with anchoring vinculin (Fig. 4b). We also demonstrated that IL-1β blockage reduced actin and vinculin synthesis (Fig. 4c). Similarly, LPS-induced cell synchronization was fully abrogated by the addition of Anakinra or αIL-1β as evidenced by the LY diffusion through the cell layer (Fig. 4d,e, Supplemental Fig. 1b).

Taken altogether, these data demonstrate that, in co-culture of macrophages and myocytes, LPS-induced secretion of IL-1β is necessary but not sufficient to induce full differentiation of myometrial cells and therefore contractions.

### During labor, macrophages play a pivotal role by secreting IL-1β

In a next set of experiments, we wanted to further characterize which cell type was responsible for the observed effect of IL-1β on labor onset.

**Figure 5:**
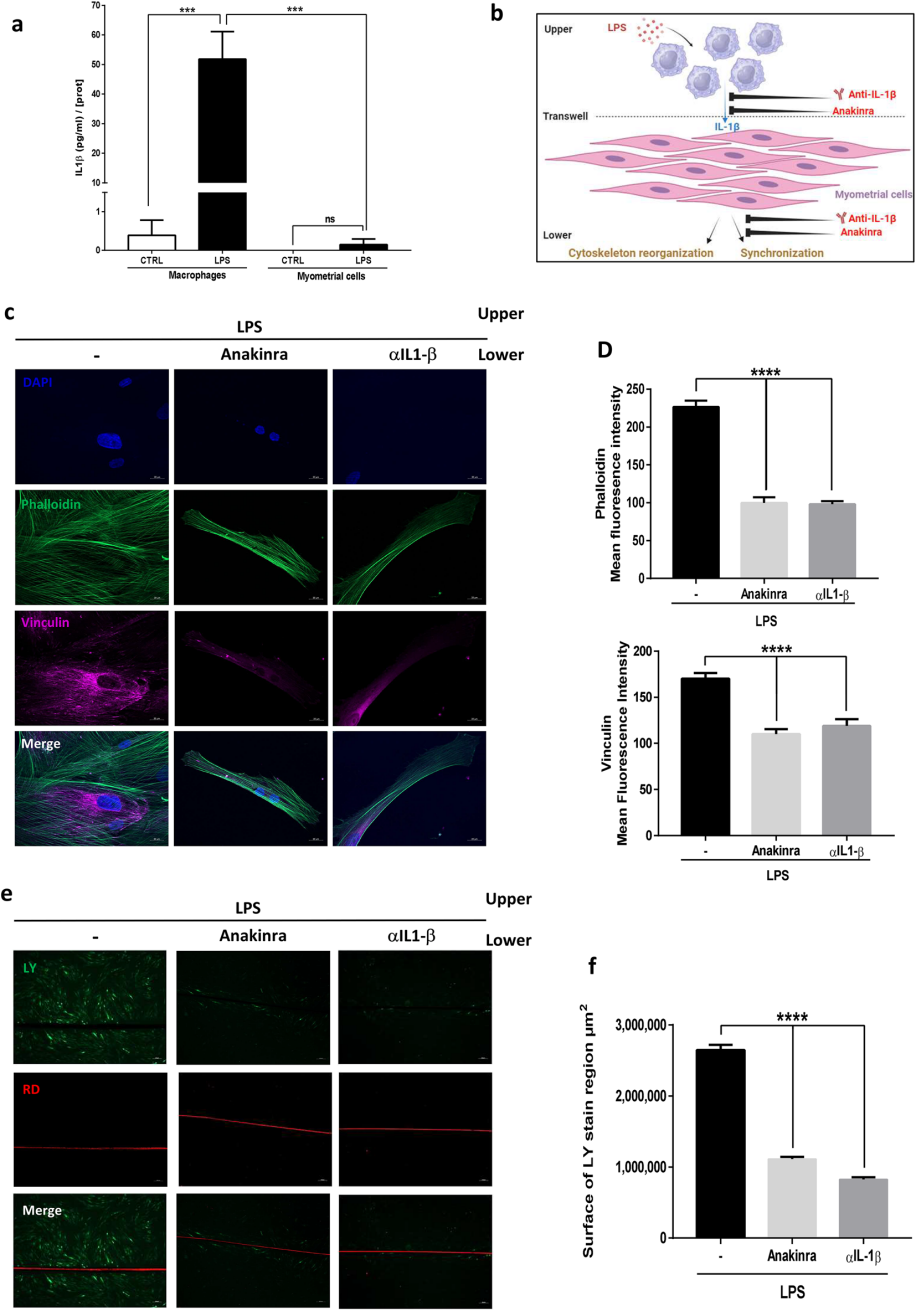
Macrophages play a pivotal role in LPS-induced differentiation by secreting IL-1β. (**a**) IL-1β concentration in macrophages or myometrial cells stimulation supernatants. Macrophages or myocytes were cultured for 24 h in presence or absence of LPS (100 ng/ml). Supernatants were collected and IL-1β concentrations were evaluated with ELISA assay and standardized with protein cell content ± SEM, n = 6, *** P < 0.001. (**b**) Schematic representation of the effects of IL-1β blockage for each cell type on differentiation of myometrial cells in a Transwell assay. (**c**) Transwell experiments with macrophages (upper compartment) and myometrial cells (lower compartment) following treatment with LPS (100 ng/ml) in presence or absence of Anakinra (1 µg/ml) or αIL-1β (100 ng/ml). Representative images of phalloidin (green), vinculin (purple) and DAPI for nuclear localization (blue) were taken with an Axiozoom epifluorescence microscope (×400) in five random chosen fields of four separated experiments. (**d**) Mean fluorescence intensity of phalloidin or vinculin ± SEM, n = 20, ****P < 0.0001. (**e**) Fluorescence images of LY (green) and RD (red) transfer to adjacent cells after scrap loading. Cells were treated for 24 h with LPS in the upper compartment and with Anakinra or αIL-1β in the lower compartment. Pictures were taken with an inverted microscope at ×100 magnification in two random chosen fields and are representative of three experiments. (**f**) Mean surface of LY staining ± SEM, n = 6, ****P < 0.0001.

First, we assessed IL-1β secretion in the supernatant of both macrophages and myometrial cells after LPS stimulation, to identify the main source of IL-1β in our model. We observed that primed macrophages, unlike myometrial cells, secreted high level of IL-1β (Fig. 5a) demonstrating that LPS-induced IL-1β secretion is mainly conducted by macrophages.

Then, we performed Transwell assays to decipher the contribution of each cell type on myometrial cell differentiation. These assays allowed us to stimulate only one cell type and to block IL-1β receptor or sequester IL-1β in the upper (macrophages) and/or lower (myocytes) compartment (Fig. 5b). LPS stimulation on macrophages (i.e., in the upper compartment) induced cytoskeleton reorganization of myometrial cells seeded in the lower compartment. Furthermore, quantification of phalloidin and vinculin staining showed that LPS induced in macrophages the expression of these proteins (Fig. 5d).

As shown in Fig. 5c, the addition of Anakinra or αIL-1β to the lower compartment prevents the cytoskeleton reorganization of myometrial cells induced by LPS stimulation of macrophages. Indeed, quantification of phalloidin and vinculin staining showed that the inhibition of IL-1β action decreased the expression of these cytoskeleton proteins (Fig. 5d). Similar results were observed when Anakinra or αIL-1β was added to the upper or both compartments (data not shown). The same observations were made when assessing cell synchronization using SL/DT assay on confluent cell layer (Fig. 5e,f, Supplemental Fig. 1c). Here, we demonstrated that neutralizing IL-1β in the lower compartment of Transwell with both Anakinra or αIL-1β prevented cell synchronization observed after LPS treatment of macrophages (i.e. upper compartment).

These results indicate that LPS-induced IL-1β secretion by macrophages is responsible of myometrial laboring-shape profile and synchronization.

### Inhibition of the NLRP3 inflammasome prevents labor onset

**Figure 6:**
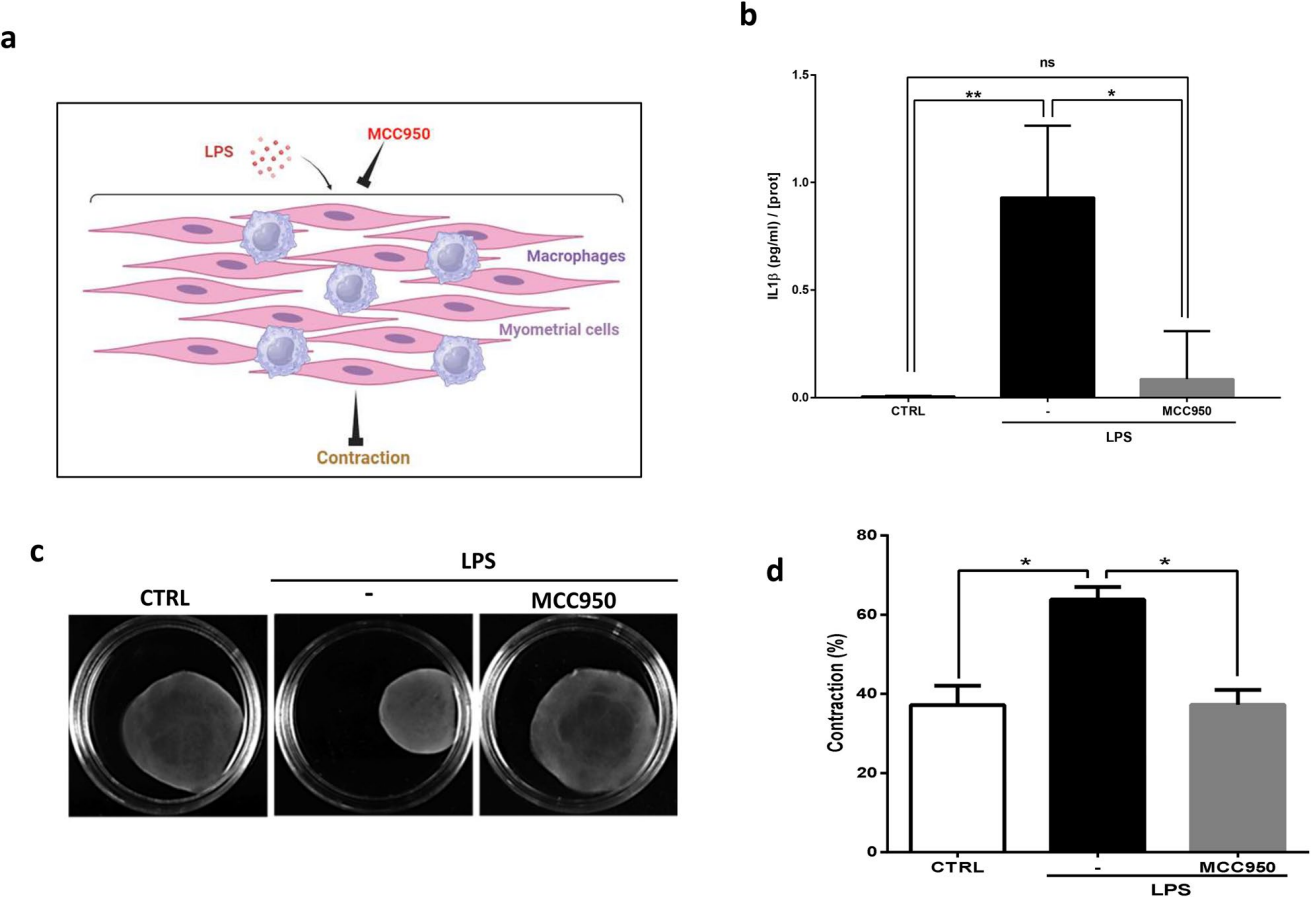
Inhibition of NLRP3 inflammasome prevents LPS-induced contractions of collagen lattices of primary human myometrial cells co-cultured with macrophages. (**a**) Schematic representation of the effects of NLRP3 inflammasome inhibition on differentiation of myometrial cells in a co-culture model. (**b**) Macrophages and myometrial cells were co-cultured for 24 h in presence or absence of LPS (100 ng/ml) ± MCC950 (0.1 µM). Supernatants were collected and IL-1β concentrations were evaluated with ELISA assay and standardized with protein cell content ± SEM, n = 6, *P < 0.05 and **P < 0.01. (**c**) Representative photographs of collagen lattices of myometrial cells co-cultured with macrophages stimulated with LPS (100 ng/ml) ± MCC950 for 96 h. (**d**) Mean percentage contraction of collagen lattices after 96 h of stimulation with LPS in presence or absence of MCC950 compared to the basal surface area, mean ± SEM, n = 5, *P < 0.05.

To be biologically active, IL-1β needs to be processed by the inflammasome that cleaves pro-IL-1β into its secreted active form. We investigated the role of NLRP3 inflammasome in our in vitro co-culture model of LPS-induced labor onset (Fig. 6a). We first confirmed the effect of the chemical inhibition of NLRP3 with MCC950 on secreted IL-1β. We demonstrated that MCC950 treatment abolished IL-1β secretion on co-cultured cells and that IL-1β level was similar to that observed in the control condition (Fig. 6b).

Then, we investigated if NLRP3 inflammasome inhibition was able to block in vitro LPS-induced labor-associated mechanisms. Indeed, as demonstrated in Fig. 6c,d, MCC950 prevented in vitro LPS-induced myocyte contraction.

**Figure 7:**
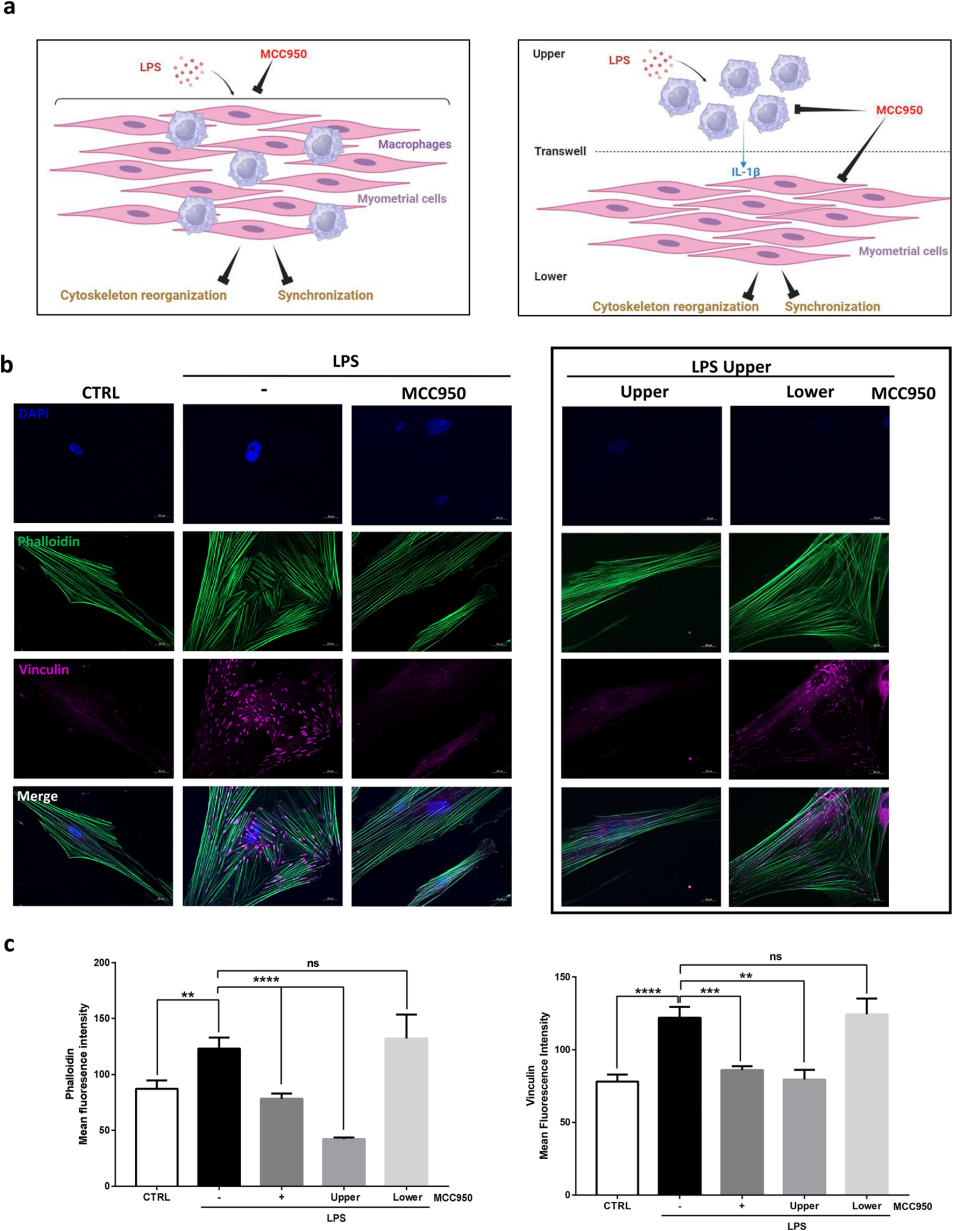
Inhibition of the macrophages’ inflammasome blocks LPS-induced remodeling of primary human myometrial smooth muscle cells. (**a**) Schematic representation of the effects of inflammasome inhibition on myometrial cell differentiation either in a co-culture model (left panel) or in a Transwell assay (right panel). (**b**) Cells were co-cultured and treated with LPS in presence or absence of MCC950. In Transwell experiments, LPS was added on macrophages (upper compartment) whereas MCC950 was added either in the upper (macrophages) or the lower compartment (myocytes). Representative pictures of the phalloidin (green), vinculin (purple) and DAPI for nuclear localization (blue) were taken with an Axiozoom epifluorescence microscope in five random chosen fields of four separated experiments. (**c**) Mean fluorescence intensity of phalloidin or vinculin ± SEM, n = 20, *P < 0.05, **P < 0.01, ****P < 0.0001.

Finally, we investigated the role of the inflammasome on myometrial cells differentiation (Fig. 7a, left panel) and the contribution of each cell type with Transwell experiments (Fig. 7a, right panel). First, co-culture was treated with LPS in presence or not of MCC950 and myometrial cytoskeleton reorganization was evaluated (Fig. 7b, left panel). Herein, we demonstrated that NLRP3 inhibition prevents myocytes remodeling and reduces phalloidin and vinculin synthesis previously induced by LPS (Fig. 7c). To assess which cell type was responsible of this effect, we evaluated NLRP3 expression in both cell types and showed that NLRP3 was mainly expressed in macrophages and that its expression was even higher after LPS treatment (data not shown). Transwell experiments with macrophages (upper compartment) and myometrial cells (lower compartment) showed that the addition of MCC950 only to the upper compartment abolished cytoskeleton reorganization (Fig. 7b, right panel) and LPS-induced over-expression of phalloidin and vinculin (Fig. 7c).

**Figure 8:**
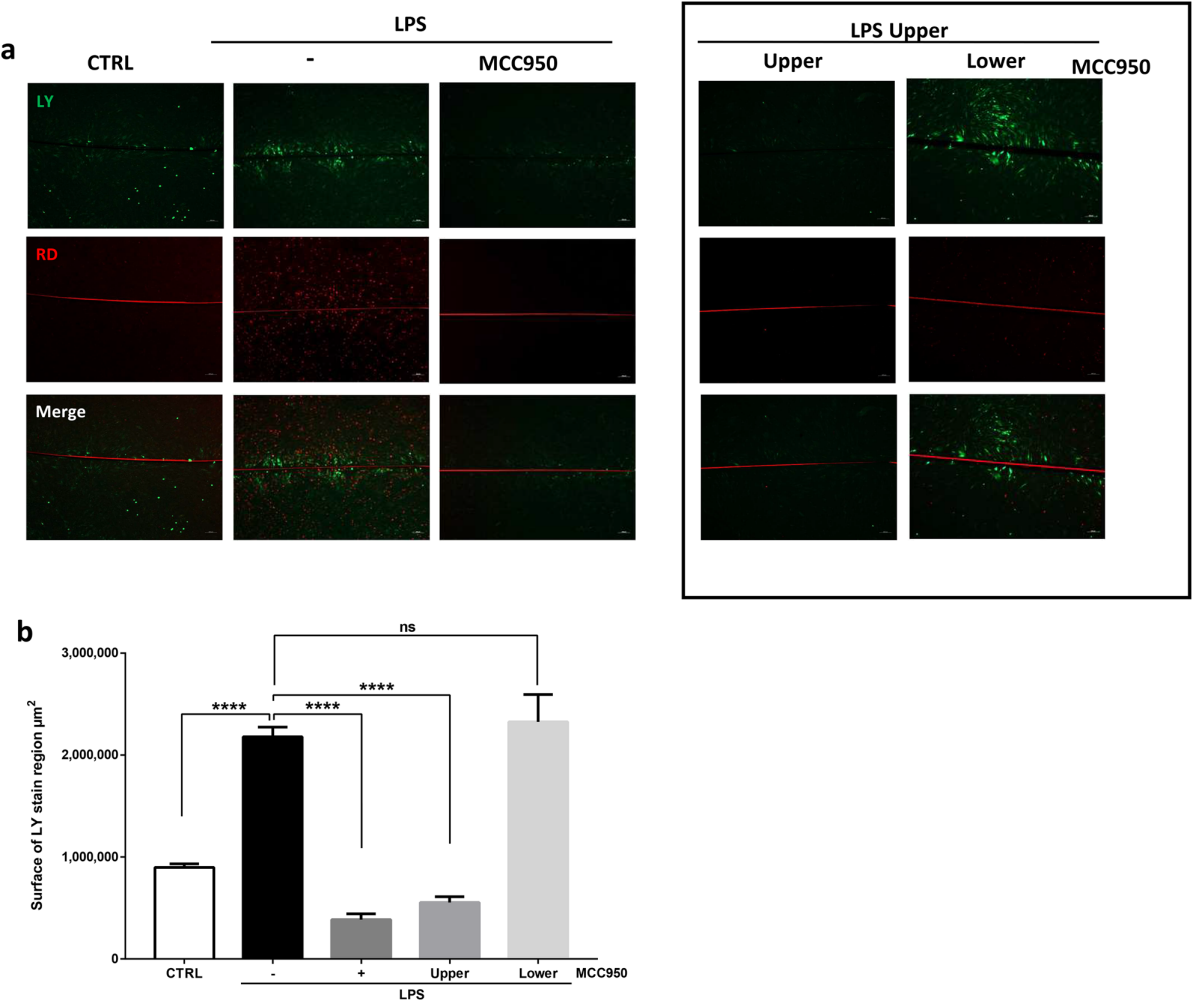
Inhibition of the macrophages’ inflammasome blocks LPS-induced myometrial synchronization. (**a**) Fluorescence images of LY (green) and RD (red) transfer to adjacent cells after scrap loading. Cells were treated for 24 h with LPS in the upper compartment and with Anakinra or αIL-1β in the lower compartment. Pictures were taken with an inverted microscope at ×100 magnification in two random chosen fields and are representative of three experiments. (**b**) Mean surface of LY staining ± SEM, n = 6, ****P < 0.0001.

Similar results were observed when assessing cell synchronization using SL/DT assay on confluent cell layer (Supplemental Fig. 1d). Indeed, in presence of MCC950, LY was unable to transfer to adjacent cells indicating that inflammasome activation was required for cell synchronization (Fig. 8a, left panel b). When cells were cultured separately, we showed that only the inhibition of the inflammasome in the macrophages (MCC950 upper) improved LY surface staining and that MCC950 treatment of myometrial cells (MCC950 lower) failed to reduced LY surface staining (Fig. 8A, right panel b).

Taken together, these results suggest that the inhibition of macrophages IL-1β secretion by MCC950 prevents the differentiation steps associated with labor onset. These data revealed the pivotal role of macrophages NLRP3 inflammasome in LPS-induced myometrial differentiation and its potential interest as a target in the pharmacological management of preterm labor.

## Discussion

Spontaneous term and preterm labor are associated with inflammation, characterized by both leukocytes’ infiltration and cytokines production in uterine tissue^5^. Among proinflammatory cytokines, IL-1β is one of the most important cytokines since non-human primates injected with a single dose of IL-1β all delivered within 48 h, while repeated injections of other cytokines are required to reproduce the same result^25^. Other studies investigated the effect of IL-1β administration on murine models and also concluded that IL-1β induces preterm birth^24,39^. Human studies reported over-expression of IL-1β in the plasma and in the amniotic fluid of term and preterm laboring women^40^. In addition, we demonstrated that IL-1β was also overexpressed in human myometrium as previously observed in rodents or in vitro.

The accumulation of these evidences makes IL-1β an attractive target for understanding labor induction^23,32^. Unfortunately, none of these studies provided a clarification in the mechanism that may explain the contribution of IL-1β on labor onset. In this study, we demonstrated that IL-1β directly induced myometrial differentiation characterized by the cells’ triangular shape. IL-1β also generated efficient contraction in a co-cultured model. These effects were prevented when Anakinra or anti-IL-1β antibody were administered.

This result also brings an explanation to discrepant results observed between in vivo and in vitro studies. Indeed, while animals injected with IL-1β delivered within 48 h, many in vitro studies failed to show a direct contractile effect of IL-1β on myometrial cells^41^. We also observed that IL-1β was unable to induce myometrial cells contraction were cultured without macrophages. Myometrial cell remodeling, described previously, is critical to promote a switch in the phenotype of the quiescent uterus to a smooth muscle, which becomes spontaneously excitable and exhibits a high degree of cell–cell coupling, but it is not a sufficient step. The gap junctional intercellular communication between myocytes is also a critical step for effective and synchronized contractions that were not observed after IL-1β stimulation. Contrary to myometrial cells alone, myocytes co-cultured with macrophages are capable of establishing intercellular communication. IL-1β blockers prevented myometrial cells IL-1β-induced synchronization when co-cultured with macrophages.

These results were supported by gene chip analysis revealing that IL-1β induced the overexpression of 91 genes in myometrial cells (mainly associated with the regulation of inflammatory processes and remodeling), but only Cox-2 among genes directly associated with labor^42^.

In conclusion, we demonstrated that IL-1β contributes to cell differentiation and synchronization but is not sufficient to generate efficient uterine contractions.

The second major interest of this study is to better define the contribution of macrophages in labor onset. The absence of a complete response of IL-1β on myometrial cells differentiation led us to investigate the NRLP3 inflammasome, an upstream actor. This hypothesis is consistent with previous studies showing that NLRP3 inhibition by MCC950 or *nlrp3* depletion showed a resistance to LPS- or S100B-induced PTL^33,34,43,44^. Here, we confirmed that MCC950 stimulation of myocytes/macrophages co-cultures blocked NLRP3 inflammasome activation, as evidenced by the drastic decrease in IL-1β in the supernatants. Furthermore, we demonstrated that NLRP3 inhibition was able to block myometrial cells contractions and cytoskeleton reorganization and synchronization. Our findings on differentiation steps expanded that of Motomura et al., that showed that uterine tissues of *nlrp3* KO mice did not exhibit increased expression of contraction‐associated proteins such as connexin-43 and oxytocin receptor upon LPS injection^33^.

Of particular interest, Transwell assays allowed us to study myometrial differentiation by specifically blocking the inflammasome in macrophages or in myocytes. Notably, we demonstrated that macrophages’ NLRP3 inflammasome was involved in myometrial contraction and differentiation. Contrary to Lappas et al.^36^ who worked on human myometrial explants, here with our co-culture models we are able to determine that macrophages’ NLRP3 inflammasome is an actor of labor-associated mechanisms. This is supported by previous studies indicating that *nrlp3* deficiency in mice dysregulates the function of uterine-infiltrating macrophages^33,34^.

**Figure 9:**
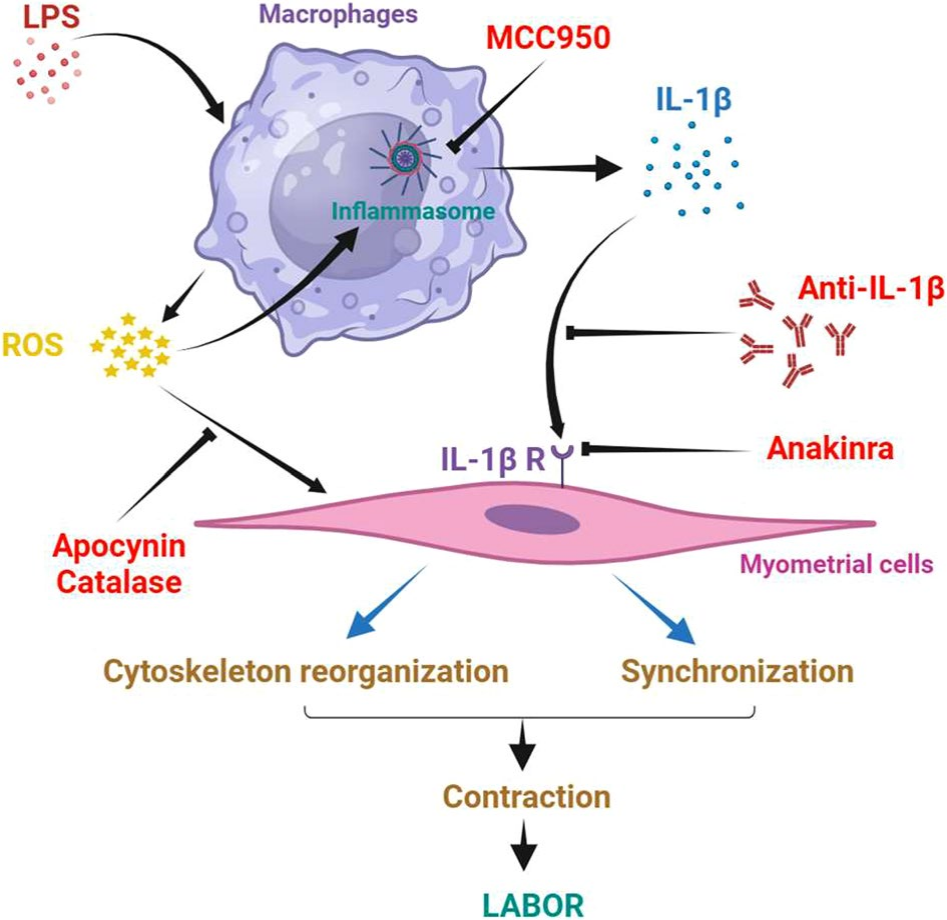
Schematic representation of putative interactions between myometrial smooth muscle cells and macrophages. This representation was a synthesis of the data presented in this article and previous publications from our team: Hadi et al*.*^12^ and Wendremaire et al.^13^. LPS treatment of myometrial and macrophages co-culture leads to the production by macrophages of ROS which contributed to inflammasome activation and IL-1β processing. Myometrial cytoskeleton reorganization and synchronization is directly achieved by IL-1β and ROS.

These data have allowed to understand better the already known pivotal role of macrophages during parturition^12,13,45,46^. Our team previously demonstrated that macrophages induced a high production of ROS, which was responsible for myometrial inflammation^12^, and that such ROS secretion promoted myometrial contraction by acting on myometrial cytoskeleton reorganization or synchronization^13^. In parallel, several studies reported that ROS induces IL-1β secretion through inflammasome activation^47–49^. The present paper provides new insights in the comprehension of the activation of macrophages in an inflammatory context and identified a new link between all known determinants of labor mechanisms, as depicted in Fig. 9.

Current tocolytic drugs (e.g., betamimetics, calcium channel blockers, oxytocin receptor antagonists) aim to delay preterm birth by targeting myometrial contractions. However, the efficacy of such treatments in terms of delaying pregnancy and improving neonatal outcomes has proven to be limited^50,51^. The limited effectiveness of these therapies is partly related to their targeting of the terminal pathway of labor onset, rather than acting on early stages. Our team and others believe that maintaining uterine quiescence seems to be a major asset in the management of PTL^52–55^. In this study, we propose a proof of concept based on the inhibition of inflammation which is responsible for the differentiation of myometrial cells. Nevertheless, blocking inflammation for PTL management is not without risk. Non-steroidal anti-inflammatory drugs such as indomethacin are associated with significant fetal effects such as constriction of the ductus arteriosus and fetal renal insufficiency^56,57^. Concerning corticosteroids, their use should be limited to fetal lung maturation when administered more than 24 h before birth, but no more than seven days before birth because of serious disorders on brain development^58,59^.

In this context, our results suggest that the selective targeting of macrophages would be an efficient way to prevent preterm labor by acting on early stages of myometrial differentiation. Our study provides also new therapeutic targets, namely IL-1β and NRLP3 inflammasome. Although IL-1β and NLRP3 inflammasome are closely linked, it appears that NLRP3 inhibition seems more effective in inflammatory-related pathologies. Nevertheless, our results were obtained in vitro and ex vivo allowing us to consider the inhibition of IL-1β or inflammasome in an in vivo model of preterm labor as potential new therapeutic targets. Thus, in vivo studies performed either with IL-1β-deficient mice^60^ or IL-1β receptor antagonists-treated mice^61,62^ failed to prevent preterm labor and perinatal brain injury. Contrariwise, Gomez-Lopez et al. demonstrated that inhibition of the NLRP3 inflammasome via MCC950 prevented preterm labor and reduced neonatal mortality, without interfering with the physiological process of term parturition^63^. Furthermore, *nlrp3* deficiency abrogated alarmin-induced preterm labor in mice, improved neonatal survival and impaired leukocyte infiltration and function in the uterus^34^. MCC950 has also shown promising results in reducing neonatal and juvenile mortality in various inflammatory diseases^64–66^. Although a study provided evidence that the treatment of inflammatory diseases with NLRP3 inhibitors is safe in humans, further research is needed to evaluate the safety of NLRP3 inflammasome inhibitors during pregnancy^67^.

## Conclusion

This study demonstrates the pivotal role of LPS-treated macrophages to transactivate myocytes through IL-1β secretion and inflammasome activation. Our results allow us to specify the implication of IL-1β and inflammasome in the early laboring stages, preceding the acquisition of a contractile phenotype. Targeting IL-1β or the inflammasome could be an interesting therapeutic opportunity in the management of preterm labor.

## Materials and methods

### Myometrial biopsies

Myometrial biopsies were obtained from pathological (chorioamnionitis, CA), normal laboring or nonlaboring (NP) pregnant women undergoing elective caesarean section between 38- and 40-weeks of pregnancy.

This study was approved by the CPP Grand Est (No. CPP: 2010/02) and the ANSM (2009-A01176-51), and performed in accordance with relevant regulations. Written informed consent was obtained from all donors.

Biopsies were cut into small strips and placed in a 24-well plate containing DMEM, incubated for 48 h at 37 °C, 5% CO_2_ and fixed with 10% formalin then embedded in paraffin.

### Chemicals and antibodies

LPS from *Escherichia coli* (serotype 055:B5; L2880), Lucifer Yellow (LY; L0259), Rhodamine Dextran (RD, D1824, *M*r = 10 kDa), human IL-1β Receptor antagonist Anakinra (SRP3327), anti-vinculin antibody (V9131), Transwell inserts with 0.4 μm pore diameter (10482181) were purchased from Sigma-Aldrich (Saint Louis, MO, USA). Granulocyte macrophage-colony stimulating factor (GM-CSF; 130-095-372) was obtained from Miltenyi Biotec (Paris, France). Alexa Fluor™ 488 phalloidin (10125092) and Alexa Fluor™ Plus 555-conjugated goat anti-mouse (A32727) were purchased from ThermoFisher Scientific, Waltham, MA, USA). IL-1β neutralizing antibody (αIL-1β, mabg-hIL-1b-3) was obtained from Invivogen (San Diego, USA) and human IL-1β Quantikine ELISA KIT from R&D Systems (Abingdon, UK). All drugs were used according to the manufacturer’s instructions.

### Cell cultures

Experiments were performed on commercial human uterine smooth muscle cells from non-pregnant women (UtSMC) obtained from Lonza (CC-2562, Basel, Switzerland) and cultured in SmBM™ supplemented with SmGM™-2 Smooth Muscle cell growth medium-2 BulletKit™. Primary human monocytes were purified from peripheral blood mononuclear cells obtained from blood donor buffy coats (provided by Etablissement Français du Sang, Besançon, France) as previously described^68^. Monocytes were then differentiated into non-polarized macrophages with GM-CSF (50 ng/ml) for 10 days in Roswell Park Memorial Institute medium (RPMI, Dominique Dutscher, Brumath, France) supplemented with 10% FBS and 1% PSA.

### Stimulation protocol

Myometrial cells were seeded in 24-well plates for immunofluorescence (2.5 × 10^4^ cells per well on sterile glass coverslip). Cells were allowed to adhere and starved for 24 h in reduced-serum media Opti-MEM (ThermoFisher Scientific, Waltham, MA, USA). Finally, the medium was removed and differentiated macrophages were added at a ratio of 1:4, as previously validated^13^. Cells were stimulated either with IL-1β at 1 ng/ml or LPS at 100 ng/ml in the presence or absence of MCC950 (0.1 µM), Anakinra (1 µg/ml) or IL-1β neutralizing antibody (αIL-1β, 100 ng/ml). For Transwell experiments, myometrial cells were cultured in the lower compartment and macrophages were seeded in the upper compartment. Then, LPS (100 ng/ml) was added to the upper compartment while Anakinra (1 µg/ml), αIL-1β (100 ng/ml), or MCC950 (0.1 µM) were added either to the upper or lower or both compartments. For each experiment, time matched vehicle controls (with the same solvent of the stock solution of the drug used for the stimulations) were performed.

### ELISA assay

Myometrial cells and macrophages were cultured alone (respectively 3 × 10^5^ or 1 × 10^6^ cells) or in co-cultures (3 × 10^5^ myocytes and 7.5 × 10^4^ macrophages) in 6 well plates. Cells were stimulated with LPS (100 ng/ml) in presence or absence of MCC950 (0.1 µM) for 24 h. Supernatants were collected and IL-1β was measured with the human IL-1β Quantikine ELISA KIT from R&D Systems according to manufacter’s instructions. Protein content was also evaluated with lowry method after cell lysis to standardize the IL-1β measure.

### Collagen lattices

Myocytes with macrophages were included in collagen gels as previously described^69^. A rat tail type-I collagen solution (3 mg/ml in 0.1N HCl, ThermoFisher Scientific) was adjusted to pH 7.2 with 0.1 N NaOH. The final concentration of collagen was 1.5 mg/ml. Myocytes (2 × 10^5^) and macrophages (5 × 10^4^) were then added to the collagen solution and were incubated in culture dishes (diameter, 35 mm) for 1 h at 37 °C to allow gelling. Then 2 ml of fresh 10% FBS DMEM was added over the cell-collagen lattice. Two days later, after replacing medium with Opti-MEM, the lattices were detached from the sides of the culture dish. The cells were then stimulated with LPS (100 ng/ml) in presence or absence of MCC950 (0.1 µM), Anakinra (10 µg/ml) or αIL-1β (1µg/ml) or IL-1β (1 ng/ml) for myometrial cells alone. Images of the gels were taken before adding the test agents and then every day for up to 5 days using ChemidocTM XRS + (BioRad,Hercules, CA, USA). The areas of the lattices were measured with Image J software, and the percentage of collagen contraction was calculated by comparing the 96 h surface area to the basal surface area. The measurements were taken from at least three separate experiments.

### Immunohistofluorescence analysis

Experiments were carried out in the ImaFlow core facility (INSERM LNC-UMR1231, Dijon, France). Five-µm sections were deparaffinized and antigen retrieval was performed in 95 °C citrate buffer. Sections are left to return at room temperature and unspecific binding was blocked with PBS—2% BSA—0.5% Tween 20. Primary antibody anti-IL-1β was incubated (1/50 dilution in the blocking solution) overnight at 4 °C followed by secondary antibody staining Alexa Fluor™ Plus 555-conjugated goat anti-mouse (1/1000). Slides were mounted in a ProLong™ Gold Antifade Mountant with DAPI for nuclear labeling (P36931, ThermoFisher Scientific, Waltham, MA, USA) and were observed with an Axiozoom epifluorescence microscope (ZEISS, Rueil Malmaison, France). Five representative pictures from three independent experiments were taken in random chosen fields and fluorescence was quantified by measuring mean fluorescence intensity using Zen Software (ZEISS, Rueil Malmaison, France). Results were expressed as means ± standard error of the mean (SEM) in arbitrary density units (ADU).

### Immunofluorescence analysis

Myometrial cells (2.5 × 10^4^) were plated on coverslips in 24-well dishes and treated as previously described. Cells were fixed in 4% formaldehyde for 5 min followed by an incubation in permeabilization-blocking buffer for 30 min (0.1% saponin, 3% BSA, 0.1% Tween 20 in PBS). Actin and vinculin were visualized using Alexa Fluor 488 phalloidin (1/400) and anti-vinculin (1/100) with Alexa-fluor 555-conjugated goat anti-mouse IgG antibody (1/1000) as a secondary antibody. Coverslips were rinsed twice in PBS and mounted in a ProLong™ Gold Antifade Mountant with DAPI for nuclear labeling (P36931, ThermoFisher Scientific, Waltham, MA, USA). The slides were observed with an Axiozoom epifluorescence microscope (ZEISS, Rueil Malmaison, France). Five representative pictures from four independent experiments were taken in random chosen fields and fluorescence was quantified by measuring mean fluorescence intensity using Zen Software (ZEISS, Rueil Malmaison, France). Results were expressed as means ± SEM in ADU.

### Scratch loading/dye transfer assay

Gap junction activity of control and treated cells was analyzed using the scratch loading/dye transfer method (SL/DT) as previously reported^70^. Briefly, myometrial cells were seeded in 24-well plates. If needed, macrophages were then added. Following treatment, a scratch was done on cell layer in the presence of LY (0.05%) and RD (2.5 mg/ml), and then incubated for 3 min at 37 °C. Cells were rinsed three times with PBS and incubated another 5 min at 37 °C before being fixed in 4% PFA. LY and RD fluorescence was visualized and measured with an Axioscope system (Zeiss, Rueil Malmaison, France). RD was used as a control remaining confined to the wounded cells bordering the scratch, whereas LY can spread to adjacent cells via open GJ channels. For each treatment, three experiments were carried out and four representative pictures were taken for each experiment. Pictures were then analyzed using Zen Software (ZEISS, Rueil Malmaison, France). Surface of LY diffusion was measured as previously described^70^.

### Statistical analysis

Statistical analysis was carried out using GraphPad Instat software (San Diego, CA, USA). Data were submitted to normality test after removing outliers according to rout method. Then, the differences between three or more groups were determined by one-way ANOVA followed by Bonferroni’s or Dunn’s multiple comparison test. Statistically significant results between groups were defined as P < 0.05 (∗), P < 0.01 (∗∗), P < 0.001 (∗∗∗) and P < 0.0001 (∗∗∗∗).

### Supplementary Information

Supplementary Information.

## Data Availability

All data generated or analyzed during the current study are included in this published article.
