# Supplementary material for: A pivotal role for the IL-1β and the inflammasome in preterm labor
Source: Sci Rep. 2024 Feb 20;14:4234. doi: 10.1038/s41598-024-54507-w (PMC10879161; doi:10.1038/s41598-024-54507-w)

A pivotal role for the IL-1β and the inflammasome in preterm labor.

Lopez TE, Zhang H, Bouysse E, Neiers F, Ye XY, Garrido C, Wendremaire M & Lirussi F.

Supplemental figure 1: Phase contrast images of confluent cells in the SL/DT assay

a) Contrast Phase of cell layer from figure 3d. b) Contrast Phase of cell layer from figure 4d. c) Contrast Phase of cell layer from figure 5e. d) Contrast Phase of cell layer from figure 8a.

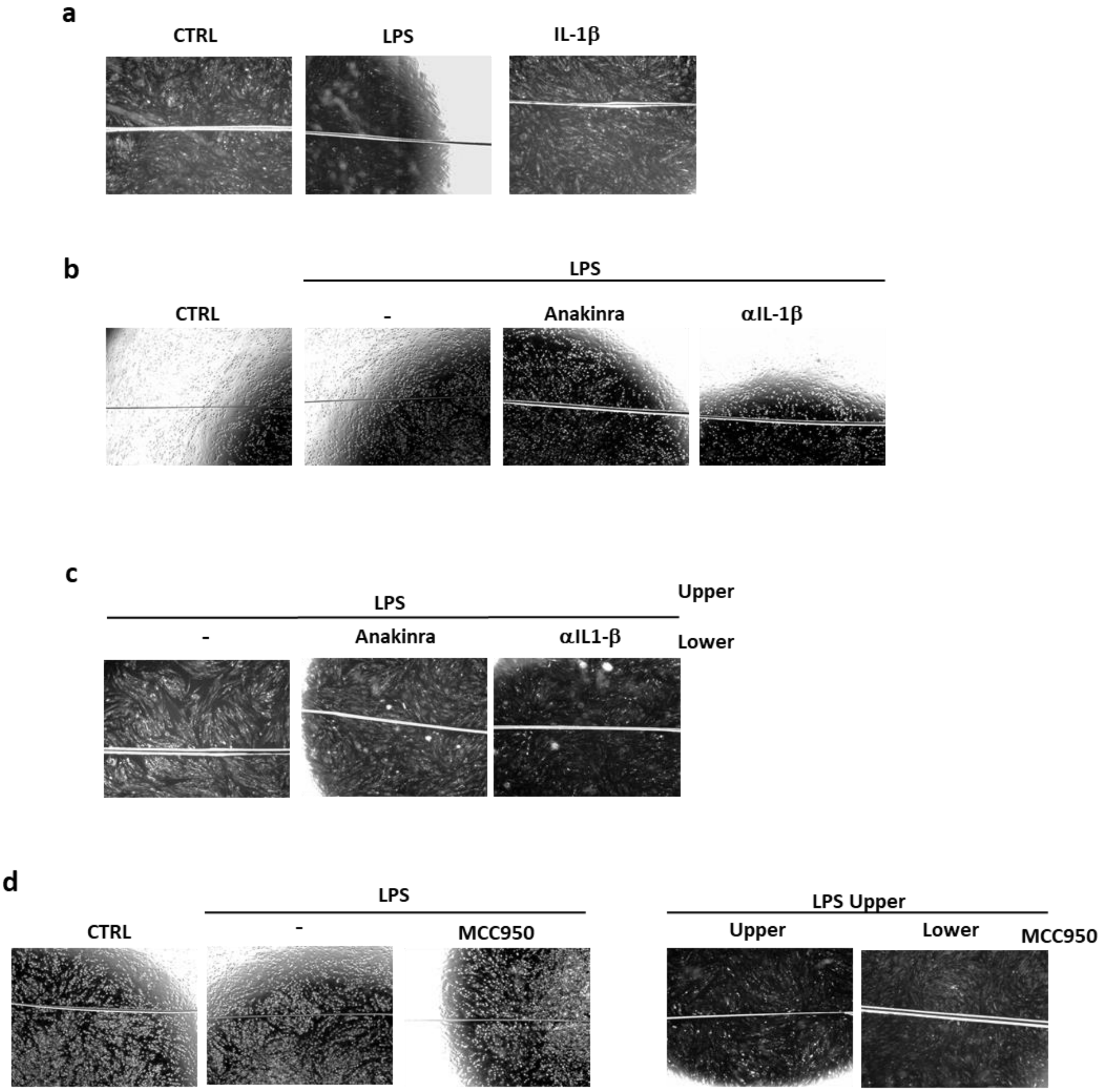

Supplemental figure 2: Phalloidin and vinculin staining on co-culture cells.

Representative images of myometrial cells and macrophages co-cultures treated with LPS (100 ng/ml) in presence or absence of Anakinra (1 µg/ml) or αIL-1β (100 ng/ml) and stained with phalloidin (green), vinculin (purple) and DAPI (blue) for nuclear localization. Images, taken with an Axiozoom epifluorescence microscope (x200), are representative of 5 random pictures of four independent experiments.

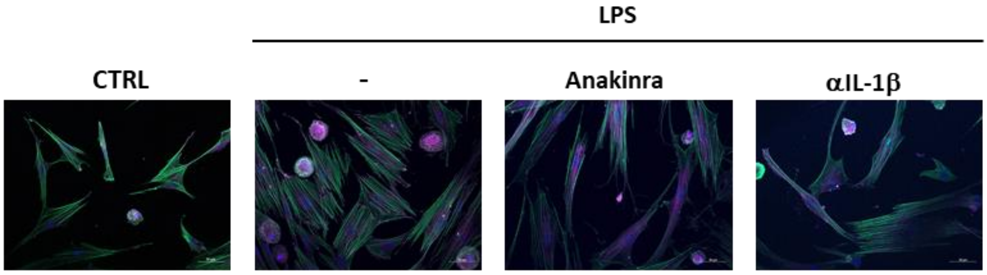

Supplement: Supplementary file 1 — Supplementary Information. [file 41598_2024_54507_MOESM1_ESM.pdf]
